# Scanning electron microscopy and machine learning reveal heterogeneity in capsular morphotypes of the human pathogen *Cryptococcus* spp.

**DOI:** 10.1038/s41598-020-59276-w

**Published:** 2020-02-11

**Authors:** William Lopes, Giuliano N. F. Cruz, Marcio L. Rodrigues, Mendeli H. Vainstein, Livia Kmetzsch, Charley C. Staats, Marilene H. Vainstein, Augusto Schrank

**Affiliations:** 1 Centro de Biotecnologia, Universidade Federal do Rio Grande do Sul, Porto Alegre, Rio Grande do Sul Brazil; 2 BiomeHub, Florianópolis, Santa Catarina Brazil; 3 Instituto Carlos Chagas, Fiocruz, Curitiba, Paraná Brazil; 4 Instituto de Microbiologia Paulo de Góes (IMPG), Universidade Federal do Rio de Janeiro (UFRJ), Rio de Janeiro, Rio de Janeiro, Brazil; 5 Departamento de Física, Instituto de Física, Universidade Federal do Rio Grande do Sul, Porto Alegre, Rio Grande do Sul Brazil

**Keywords:** Fungi, Electron microscopy

## Abstract

Phenotypic heterogeneity is an important trait for the development and survival of many microorganisms including the yeast *Cryptococcus* spp., a deadly pathogen spread worldwide. Here, we have applied scanning electron microscopy (SEM) to define four *Cryptococcus* spp. capsule morphotypes, namely Regular, Spiky, Bald, and Phantom. These morphotypes were persistently observed in varying proportions among yeast isolates. To assess the distribution of such morphotypes we implemented an automated pipeline capable of (1) identifying potentially cell-associated objects in the SEM-derived images; (2) computing object-level features; and (3) classifying these objects into their corresponding classes. The machine learning approach used a Random Forest (RF) classifier whose overall accuracy reached 85% on the test dataset, with per-class specificity above 90%, and sensitivity between 66 and 94%. Additionally, the RF model indicates that structural and texture features, e.g., object area, eccentricity, and contrast, are most relevant for classification. The RF results agree with the observed variation in these features, consistently also with visual inspection of SEM images. Finally, our work introduces morphological variants of *Cryptococcus* spp. capsule. These can be promptly identified and characterized using computational models so that future work may unveil morphological associations with yeast virulence.

## Introduction

The methodological advances achieved during the last decade allowed the study of phenotype heterogeneity in clonal cells. Particularly, the use of microorganism models has challenged the classical view of the phenotype determinacy based on the genotype and environmental context^1–5^.

It is generally accepted that phenotypic heterogeneity leads to an increase in fitness and diversity independently of genetic mutations^6^. In this regard, microbial populations benefit from the ability to switch morphotypes and create resilient subpopulations better equipped to adapt and survive in diverse environmental and host niches. This morphotype heterogeneity is evident in a wide range of characteristics, many of which are fundamental to microbial virulence^7^.

A central requisite in such studies is the use of powerful detection methods and the application of statistics^8,9^. In particular, image analysis is a common task within the machine learning field^10,11^. Yet, the literature on classification of electron microscopy-derived images for biological applications is notably sparse. As such, most algorithmic implementations focus on simpler methods for image data generation^12^.

The human pathogenic yeasts belonging to the *Cryptococcus* complex have been consistently used as a model for the study of pathogenicity and for the development of better therapeutic approaches. Infections caused by this yeast kill 180,000 people around the world every year^13^. In addition, the treatment of cryptococcosis is unaffordable in most in developing countries^14,15^.

An important trait for cryptococcal virulence is the presence of a polysaccharide capsule, which has been well described at the molecular and functional levels^16,17^. Capsular morphology includes a huge heterogeneity among distinct isolates and even in clonal isolates. This cellular diversity has been exploited in terms of drug resistance and pathogenic potential^18–20^.

The study of phenotype heterogeneity is hampered by the lack of proper detection methods and statistical analyses. In order to further explore this morphotype diversity, we implemented an automated image analysis pipeline. This machine learning approach is capable of detecting and classifying capsular morphotypes, being applicable to cell type quantification in microscopy-based experiments. While not a high-throughput technique per se, SEM does yield vast amounts of complex data. Here, we describe the adaptation of one algorithmic implementation for the analysis and classification of *Cryptococcus* spp. capsular morphotypes captured using scanning electron microscopy (SEM). Our model substantially increases data analysis efficiency and provides a template for future machine learning applications within microbiology.

## Results

### *Cryptococcus* spp. exhibit distinct capsule morphotypes under scanning electron microscopy

**Figure 1:**
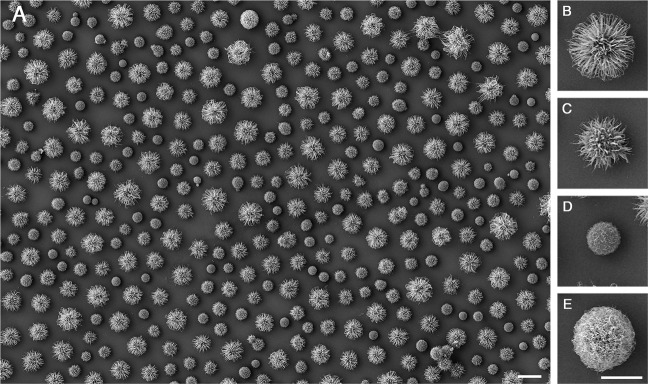
Morphological diversity of the cryptococcal capsule based on SEM analysis of a clinical isolate of *C*. *gattii*. (**A**) SEM image showing the coexistence of different morphotypes, which were classified on the basis of morphological characteristics including fiber abundance, capsule thickness, size and apparent texture. The capsular morphotypes were named regular (**B**), spiky (**C**), bald (**D**), and phantom (**E**). Scale bars: 10 μm (**A**) and 5 μm (**B**–**E**).

The analysis of *Cryptococcus* spp. morphology by SEM revealed different capsular morphotypes within clonal microbial cultures (Fig. 1A). From the visual observations, we named four different morphotypes which persistently appeared in varying proportions among different yeast isolates. These morphotypes were defined as regular (Fig. 1B), spiky (Fig. 1C), bald (Fig. 1D) and phantom (Fig. 1E).

### Image segmentation with ebimage

**Figure 2:**
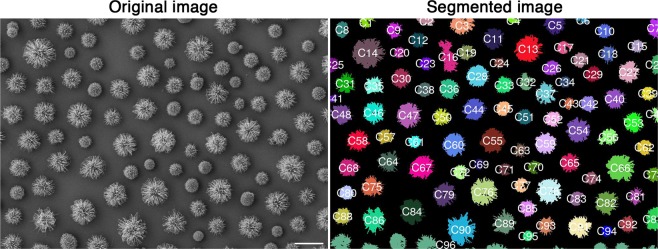
Illustration of image segmentation from a SEM image. The colors were randomly chosen to represent the distinct objects detected by EBImage. Unique identifiers are assigned to each object, and those were used for posterior manual labeling. Note that most cells are well distinguished, although marginal objects tend to be merged together, as illustrated by C96 at the very bottom. These merged cells, as well as other non-cellular objects, were all treated as artifacts. Cells that were not displayed entirely in the field of view such that no visual classification was possible were considered unidentified. Scale bar: 10 μm.

*Cryptococcus* SEM raw images (N = 11, total cells = 811) were processed using the EBImage package available from Bioconductor^12^. Shortly, the pipeline segments images based on pixel intensity spatial distributions. Background is separated from potentially cell-derived entities. Objects comprised of too few pixels were discarded, and remaining instances were visually classified as their respective cellular morphotypes, artifacts, or unidentified (cells of unknown morphology), Fig. 2.

For each identified object, features such as pixel intensity, moment (*e*.*g*. eccentricity), shape and texture were extracted using the corresponding commands from the EBImage package^12^. Over 50 features per cell were obtained, the majority of which were invariant to cell position and/or rotation.

### Visualizing overall profiles with principal component analysis

The final data matrix obtained was composed of over 800 cells (rows) and over 50 features (columns). In order to verify whether there were clear groupings among the observations, exploratory analysis was performed through Principal Component Analysis (PCA)^21^. This procedure achieves dimensionality reduction by projecting the data matrix into a new subspace, composed of linearly independent vectors (Principal Components – PCs), which can be treated as new, uncorrelated features. These new features are ordered decreasingly in terms of total explained variance and hence can be used to visualize global tendencies of the original data in lower dimensional space.

**Figure 3:**
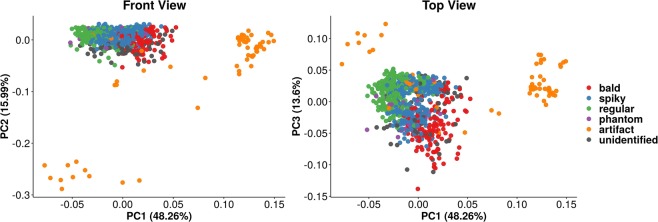
PCA of SEM image data. Each point represents a single cell, each color represents a given label: bald (red), regular (green), spiky (blue), phantom (purple), artifact (orange) or unidentified (grey). The left chart shows PC1 and PC2, resembling a “front view” of PCA. Bald, spiky, and regular cells show grouping patterns, although artifacts differ greatly. Unassigned cells appeared well distributed all over labeled cells, which indicates they may belong to any morphotype. The right chart shows a “top view”, confirming the previous observations.

Figure 3 shows the PCA for the SEM images. Each axis is a Principal Component (PC) with its corresponding proportion of explained variance. Each point in the plot represents a single cell, and the colors represent the cell morphotypes. Points that lay closer together have similar overall profiles across all original features, while distant observations in the chart tend to be distinct in nature. The data captured the differences among the capsular morphotypes, although clustering is hindered by partial overlap. This was further tested using a non-parametric version of multivariate ANOVA, which is capable of testing whether groups of observations show significantly different centroids^22^. Considering only those objects with actual cell morphotype labels, PERMANOVA revealed significant overall differences across cell types (P < 0.001). Also, the artifact observations were placed distant from all the labeled cells, indicating that their profiles differed in great extent. Finally, unidentified objects appeared distributed throughout the regions populated by labeled cells, which suggests they display cell-like properties and probably correspond to actual cells that failed to be manually classified into their corresponding morphotypes.

### Cellular morphotypes significantly differ in shape, moment, pixel intensity, and texture

**Figure 4:**
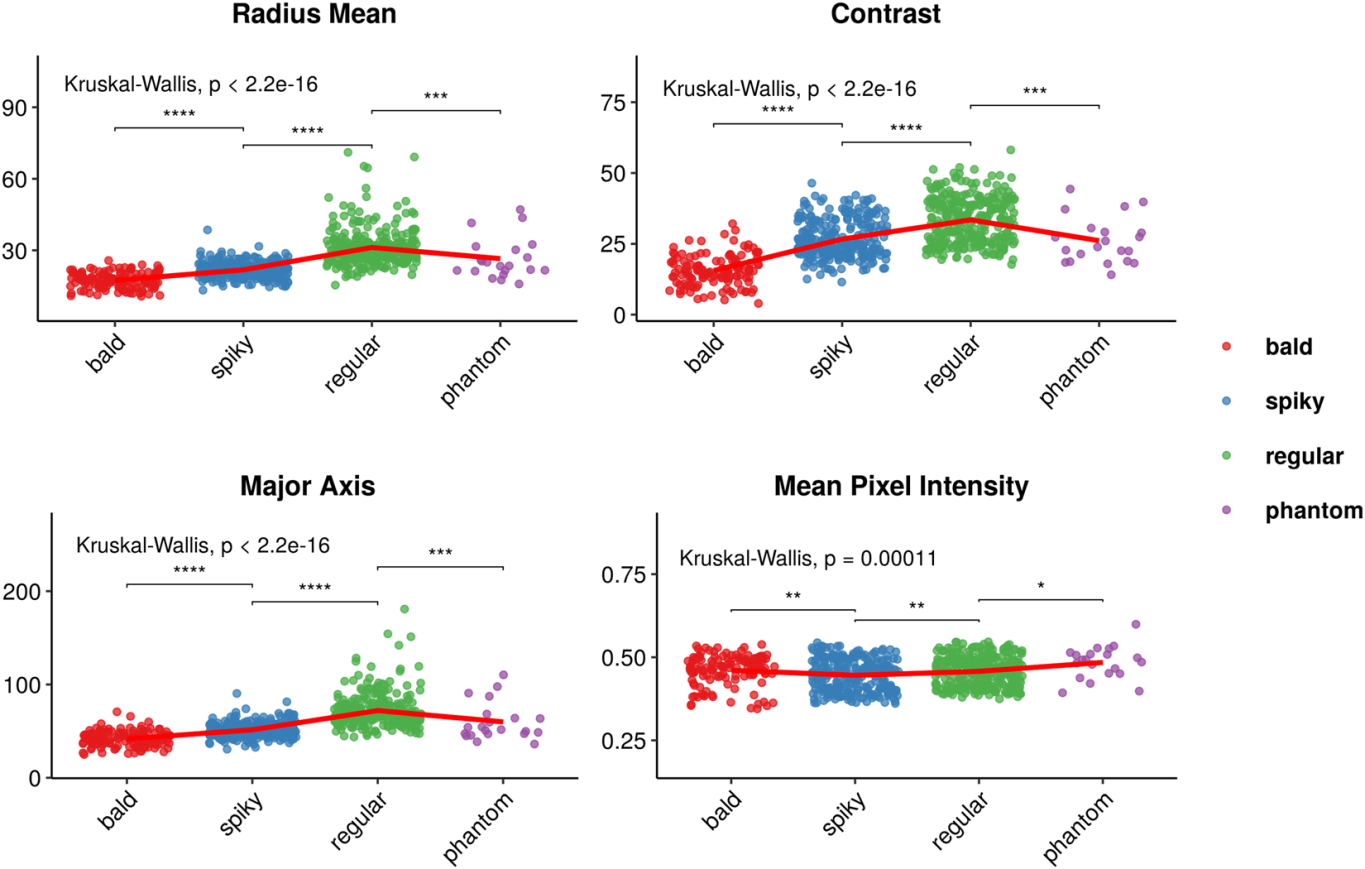
Capsular morphotypes differ significantly in mean radius, major axis size, mean pixel intensity, and contrast. Each point represents a single cell, and a small noise was added to each position to avoid overlap. Differences in shape (mean radius) tend to correlate with differences in moments (major axis), but also with texture (contrast). Pixel intensities (basic feature) differed significantly, but effect sizes do not seem to be nearly as large. The types of the features are named as defined in the EBImage R package. Red lines connect the means. Overall and pairwise comparisons computed with Kruskal-Wallis and Wilcoxon tests, respectively. ^*^p < = 0.05; ^**^p < = 0.01; ^***^p < = 0.001; ^****^p < = 0.0001.

To further investigate morphological differences across identified cells, we used robust methodologies to compare basic (pixel-intensity related), shape, moment (eccentricity), and texture features across morphotypes. Figure 4 illustrates how the cells differ. The red lines connect the means, and pairwise comparisons were performed using Wilcoxon test. Although significant differences were often observed, note how pixel intensities do not seem to differ as much as contrast. Not surprisingly, mean radius seems to vary coordinately with major axis size.

**Figure 5:**
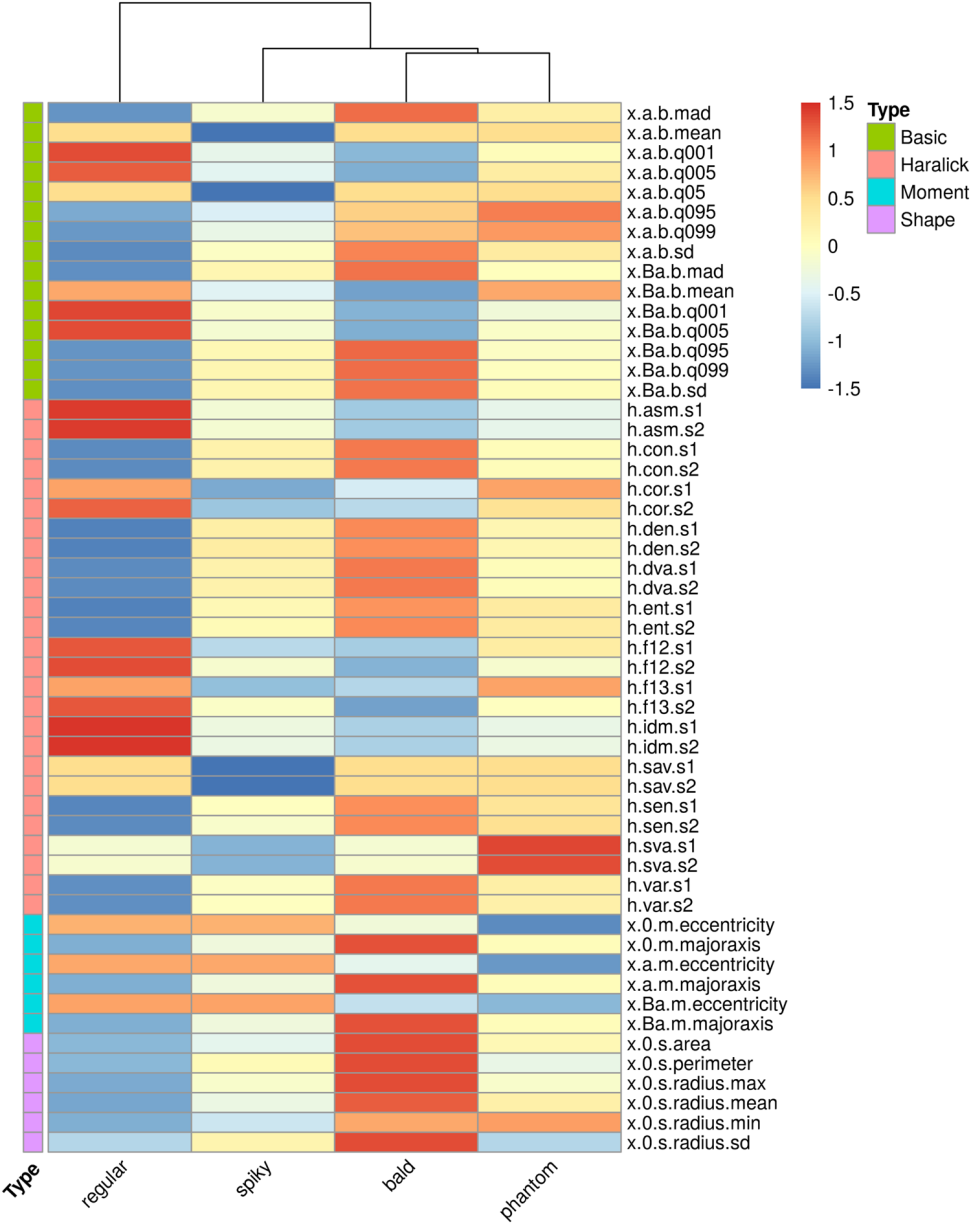
Capsular morphotypes differ significantly in intensity, shape, moment, and texture. We estimated the expected value (mean) for each cell morphotype across all features using robust regression. The scaled values are represented as color intensities, i.e., the greater the intensity, the greater a given cell type differs from the cross-group average for the corresponding feature. Non-significant regression coefficients were set to zero and in these cases the resulting color corresponds to the baseline (estimate for regular morphotypes). Near-zero values mean the cell morphotype was close to the cross-group average.

As there were over 50 features to compare, we adapted a common analysis strategy from the genomics literature. Using robust regression, we estimated the means of each cell morphotype for all features. After multiple-comparison correction of P- values, non-significant regression coefficients were set to zero and contrasts were used to compose the estimated expectations. Translation- and rotation-variant features were left aside, as these represent positioning rather than cell type morphology. The scaled estimates were used to construct the heatmap shown in Fig. 5. We observed greater differences among shape and Haralick features, suggesting shape and texture are major components of morphological differentiation, consistent with visual inspection.

### Machine learning predicts cellular morphotype

In order to assess the degree with which the identified morphotypes were differentiable, we performed supervised classification using a Random Forest (RF). We split the cells into training and test sets with representative proportions of target labels (capsular morphotypes). Seventy five percent of the data were assigned to the training set. Using repeated 10-fold cross validation, we applied the Rborist algorithm (from within the Caret R package interface) to perform grid search on hyperparameters that were relevant for classification optimization^23,24^. The optimized parameters included the number of randomly selected predictors considered at each split within each tree (known as *mtry* or *predFixed*) and the minimal node size (*minNode*) considered for all trees in the RF model.

At this stage, the random forest was built of over 2,000 decision trees. Each tree splits the data into recursive partitions in a way that minimizes a function of node impurity, often the Gini index or Entropy^10^. By considering an aleatory subset of predictors at each split (“random”), the algorithm yields many uncorrelated trees. Among this aggregation of trees (“forest”), the majority vote for each observation is taken as its actual class. Supplementary Fig. S1 depicts the classification performance as a function of the number of randomly selected predictors for 10 different values of minimum node size. Using the training set, the top-performing hyperparameter values observed were 11 randomly selected predictors at each split and 7 as the minimum node size.

**Figure 6:**
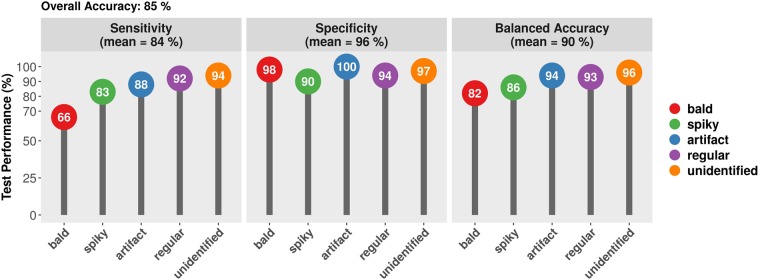
Random Forest prediction performance. Bald (red), spiky (green), regular (blue), artifact (purple) or unidentified (orange) morphotypes are shown. Performance values were sensitivity (left panel), specificity (middle panel), and average (balanced accuracy, right panel).

Once the RF classifier was built, we used it to predict the morphotype classes in the test set. This included the three cell types (bald, regular, and spiky), as well as artifacts and unassigned (unidentified) cells. This approach allowed for cell identification in the presence of unwanted objects, which are inherent to (and a current challenge for) high- throughput image segmentation^25^. Phantom cells were not included in as the sample size was considered too small for predictive modeling. The results are shown in Fig. 6. The overall accuracy was computed as the proportion of total correct predictions, settling at 85%. The model presented highly specific predictions for all classes (mean 96%, standard deviation 3.5%), while sensitivity varied more broadly (mean 86%, standard deviation 12.2%). Bald cells yielded the lowest sensitivity value (66%), while the greatest sensitivities were reported for regular cells, unidentified objects, and artifacts (93%, 94%, and 94%, respectively). All target classes showed specificities above 91%. Balanced accuracy (mean 91%, standard deviation 6.9%) was computed as the average between sensitivity and specificity for each group, ranging between 81% (bald) and 97% (artifact).

**Figure 7:**
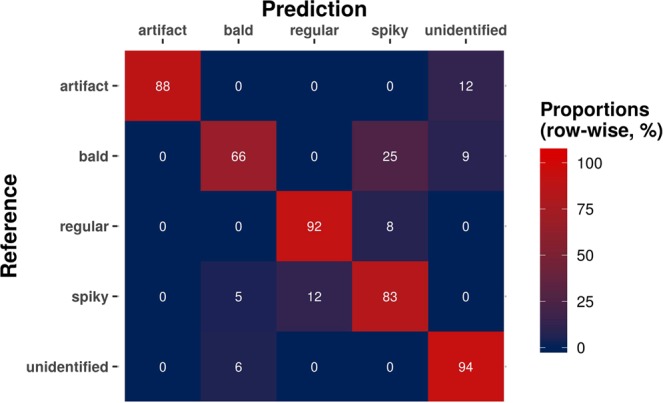
Cross-tabulation of observed and predicted values from RF classifier on the test set (confusion matrix). Proportions were calculated on a row-wise basis so that the principal diagonal shows the sensitivity values for each class, *i*.*e*., the proportion of cells labeled in each class and detected as such. For example, 92% of regular cells were correctly classified, while 8% were mistakenly predicted as spiky.

One of the main challenges for the predictive performance is the bias from manual labeling. This manual process is often affected by image-to-image variability, which itself can also be a source of bias. One way to overcome this issue is to increase the number of images analyzed. Nevertheless, such a procedure also relies on operator availability. Here, we used the so-called confusion matrix^23^, a cross-tabulation of observed and predicted classes, to observe error tendencies from our model (Fig. 7). Columns represent the predicted morphotypes and rows represent the references. The correct predictions are in the principal diagonal, which represents the sensitivity values as the numbers shown are the relative proportions from row-wise calculations.

From the confusion matrix, it was clear that many bald cells are mislabeled as spiky, and many spiky as regular. These were the main errors leading to the two lowest sensitivity values observed (66% and 83%, for bald and spiky cells, respectively). These results may be affected by biases in visual labeling as these are often misinterpreted by the human eye. Also, unwanted objects (artifacts and unidentified) were mostly detected as such.

**Figure 8:**
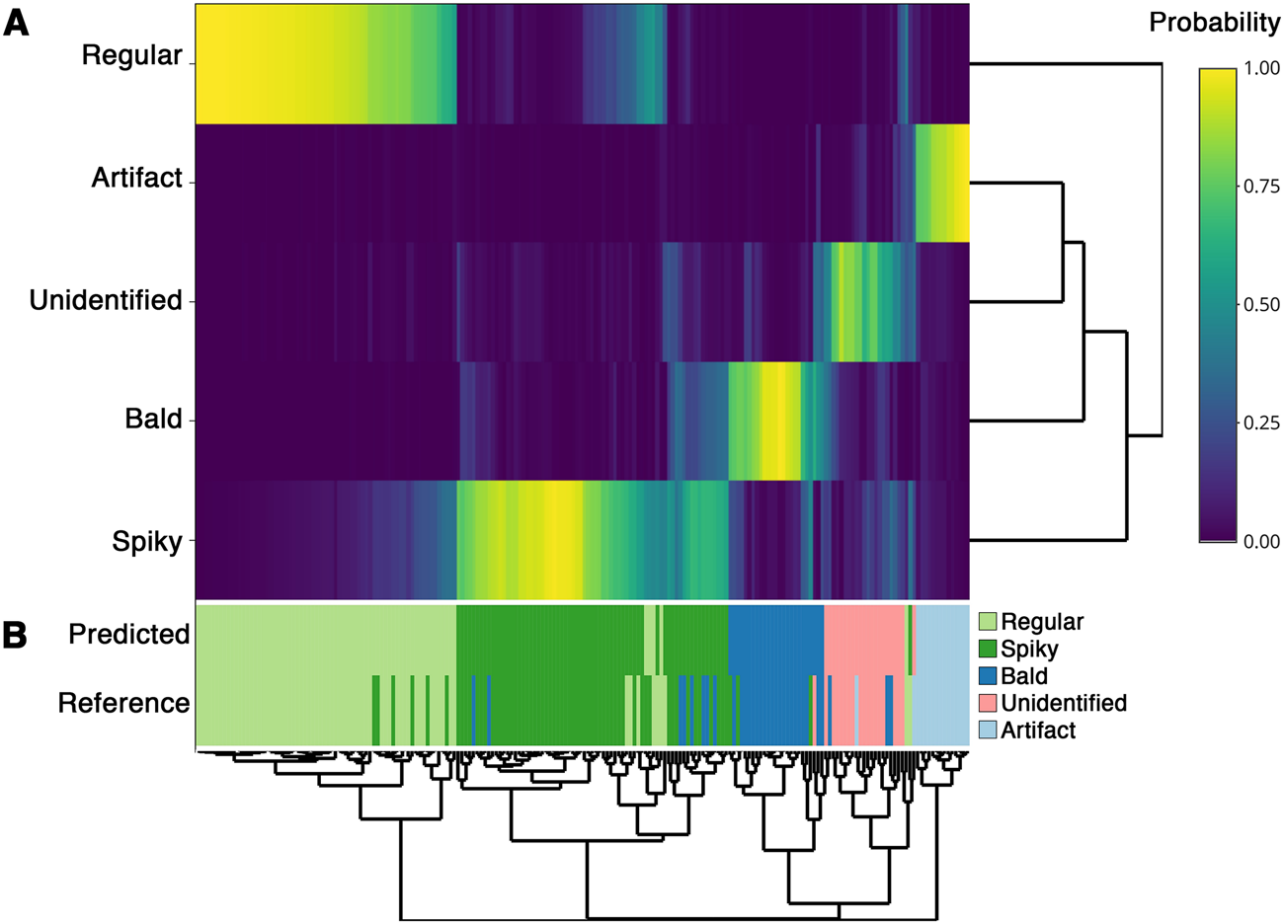
Heatmap of class probabilities obtained from the RF model. (**A**) The predicted probabilities are represented in the first five rows. (**B**) The predicted classes and reference labels are displayed on the remaining two rows. Most mismatches between predicted and reference rows (classification errors) occurred among objects difficult to differentiate even visually. This observation indicates that biases from manual labeling might be a significant challenge for classification accuracy.

To further investigate the sources of error, the same results were plotted in Fig. 8 as a heatmap of class probabilities. As expected, unequal probabilities yield mostly correct predictions, and the classification challenges exposed by the confusion matrix were generally represented by cells with similar probabilities of morphotype assignment and, therefore, difficult to visually differentiate (*e*.*g*. bald versus spiky). These results indicate that manual labeling may be a major issue in predictive modeling of *Cryptococcus* spp. morphotypes using SEM images. However, such an effect may be resolved, for instance, with greater sample sizes. Nonetheless, it was clear that the pipeline showed satisfactory performances for object detection and classification, thereby enabling automated cellular morphotype analysis in SEM-derived *Cryptococcus* spp. images.

### Image labeling with predicted morphotypes

**Figure 9:**
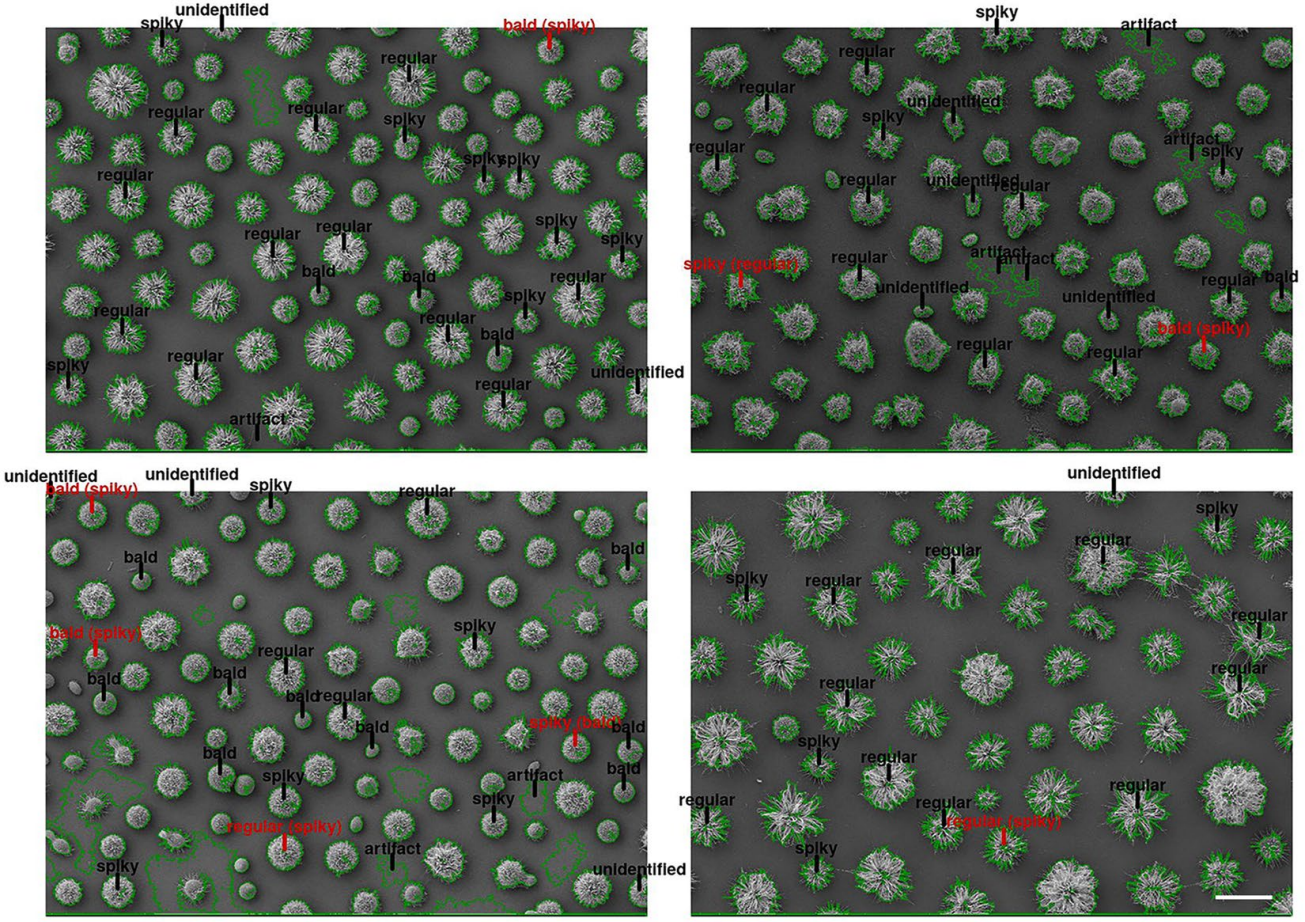
Original images with classification annotations. The predicted classes were mapped to their corresponding objects and drawn on top of the original images. Objects detected in the segmentation step are highlighted in green. Correct predictions are shown in gray, while misclassifications appear in red (with correct label in parentheses). Scale bar: 10 μm.

A drawback from random forest analysis, however, is the lack of interpretability^26^. Even though a given tree can be visualized from the resulting RF, this is not representative of the ensemble model. In order to overcome this issue, we plotted the actual predictions over the subjects of study. Figure 9 shows the original images along with the predicted annotations. The gray labels represented the correct predictions, while the red labels represented classification errors with the actual class within parentheses. The annotated objects were sparse as most observations were used for model building (comprising the training set). The visualization, hence, was constructed over the test set solely (N = 202). The green lines highlighted the detected objects from the automated segmentation procedure.

### Variable importance

**Figure 10:**
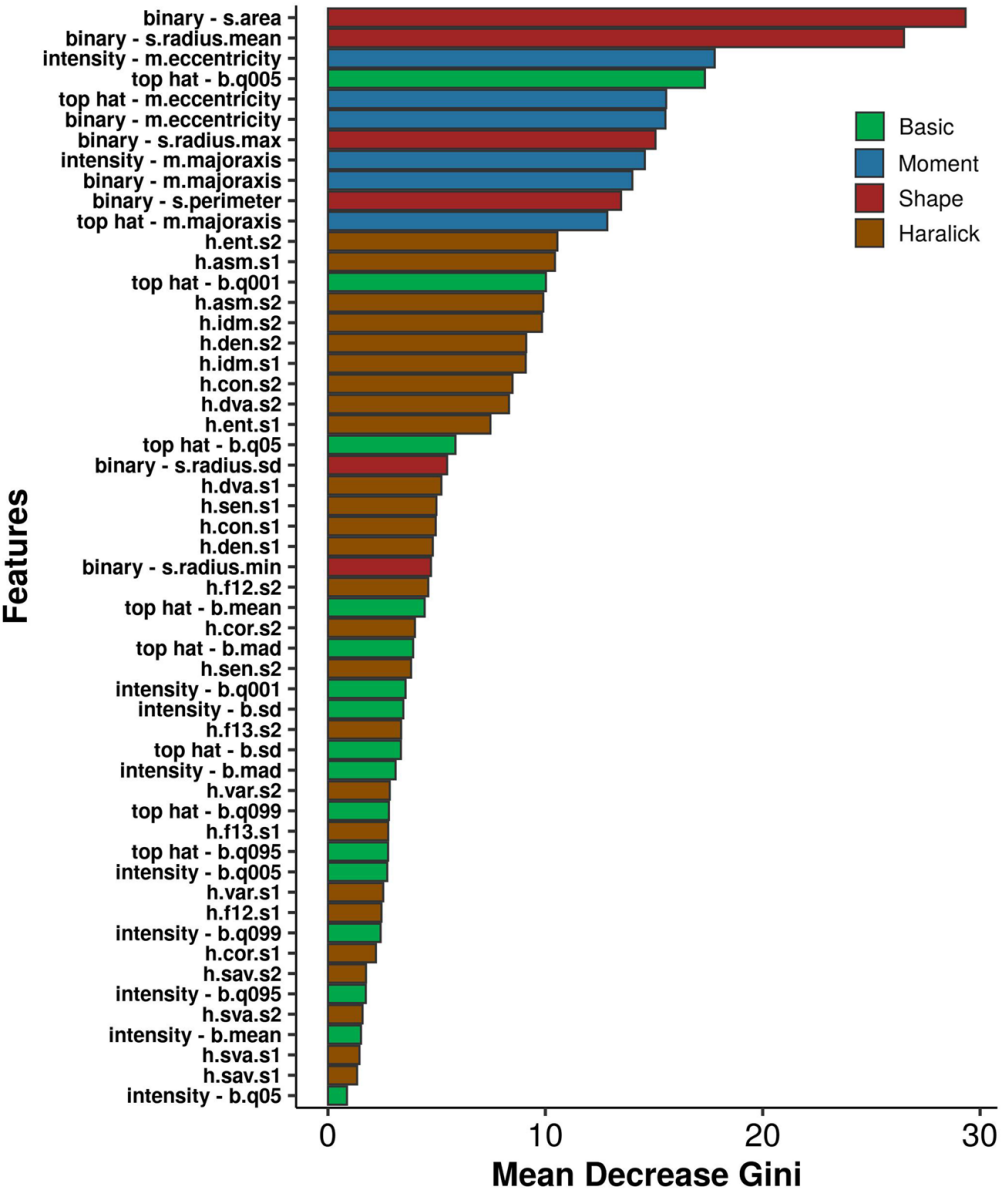
Feature importance and the mean decrease in the Gini index. Features are colored by type and may be calculated from three different sources: pixel intensities, segmentation-derived binary masks (*i*.*e*. images encoded as matrices in which identified objects are represented by 1, while background is coded as 0), and morphological top-hat transformations of pixel intensities.

A useful application of random forest classifier is to infer variable importance, i.e., to estimate how important a given feature is for the overall classification task. For instance, features that cause major decreases in the Gini index, a measurement of node impurity, tend to show higher importance. Figure 10 shows such a measurement for all features considered during model building. Feature names describe source (*e*.*g*. pixel intensities or objects’ binary masks), type (*e*.*g*. “s” for shape, “h” for haralick), and label (*e*.*g*. “radius.max” stands for maximum radius). For instance, to compute “binary - s.radius.sd”, the EBImage package uses the binary mask generated from objects’ segmentation (1 = foreground, 0 = background) and calculates the standard deviation of a set of radius measurements obtained for a given object.

Among the features with highest mean decrease in the Gini index, moment and shape features were notably prevalent - followed by Haralick features. This observation suggests that objects’ structural characteristics had the highest influence on classification. Texture measurements also showed persistently high values, while basic features were less frequent among the most relevant variables. Indeed, the 5-quantile of pixel intensities, as measured after top hat transformation (top hat – b.q005), appeared among the 5 with the greatest mean decrease in Gini index. Feature cumulative information is what builds the random forest classifier, so assessment of individual predictors would not be representative. Still, each variable may leave clues about the partitions that the many decisions trees produce at each recursive split. A Principal Coordinate Analysis was performed to yield a glimpse into the classification mechanism performed by the RF algorithm (see Supplementary Fig. S2). The actual relationships, distributions, and correlations for five of the most relevant features are detailed in Supplementary Fig. S3.

## Discussion

Image analysis is a common task in machine learning, being especially valuable for biomedical applications^25^. Most research efforts, however, have been focused on technologies effortlessly adaptable to high-throughput processing, *e*.*g*., light and fluorescence microscopy techniques^27–29^. While not as scalable, SEM produce high-resolution images for detailed phenotypic characterization at the single-cell level, shedding light onto otherwise obscure microbial diversity profiles^30^. Still, proper characterization relies on reproducible observation of cellular morphotypes, which encouraged the study herein presented.

*C*. *gattii* and *C*. *neoformans* are deadly pathogens with major mortality impacts worldwide^13^. The heterogeneity of its morphotypes has been demonstrated to be clinically relevant both in terms of virulence and drug resistance^18^. Here, we extensively characterized distinct capsule morphotypes and constructed a random forest classifier to predict such morphotypes in SEM-derived images. Based on the EBImage R package, the pipeline properly identified objects that potentially represented actual cells, differentiating these from the image background. After cell segmentation, feature extraction, and manual labeling, the cells showed significantly different shapes, moments, textures, and pixel intensity patterns. The differences detected were sufficient to yield reasonable predictions of cell subtypes using machine learning algorithm.

Model assessment revealed that class prediction was partially hampered by morphotypes difficult to differentiate visually, even for experienced operators. Nonetheless, random forest achieved satisfactory predictive performance through model tuning using grid search across relevant hyperparameters. The algorithm is known to reduce prediction variance as in bagging^26^. Although both techniques rely on ensembles of decision trees, RF de-correlates the generated trees by selecting a randomly-chosen subset of original features at each split. Suited for multi-class problems, our model was able not only to identify cell morphotypes, but also artifacts that remained from cell segmentation and even cells that could not be visually classified, *e*.*g*., cells that were only partially displayed in the image frame. Furthermore, random forest models have the advantage of generating estimates for variable importance. Our pipeline produced over 50 features of different types. In partial accordance with visual intuition, the decrease in Gini index, caused by variable permutations, indicated that structural features are most relevant for class prediction, followed by texture - although pixel intensities yielded important characteristics as well.

Finally, concerns may be risen regarding the biological meaning of the presented morphotypes. It is still unknown whether polysaccharide serotype and capsular morphotypes are correlated. However, the reported variation of capsular architecture within a single serotype of *C*. *gattii* argues against this hypothesis^31^. This highlights the importance of further investigation of capsular populations across genotypes and serotypes of *Cryptococcus* spp.

In conclusion, this study has applied computational tools to investigate clinically-relevant microbial morphotypes detected by SEM. We described *Cryptococcus* spp. capsular morphotypes in terms of shape, moments, texture, and pixel intensities. We also demonstrate these cellular morphotypes can be accurately predicted using machine learning techniques, which also gave insight into which features were most relevant for describing cell differences. It is clear that regular, bald, spiky, and phantom cells comprise significantly divergent morphotypes. It remains to be investigated, however, to which extent these cellular variations may affect virulence potential, drug susceptibility, and clinical practice.

## Materials and Methods

### Cryptococcal strains and growth conditions

A total of 11 isolates (*C*. *gattii* strains: R265, WM 161 and 7 clinical isolates; *C*. *neoformans* strains: 2 clinical isolates) were included in this study. Cells were grown for 24 h at 30 °C in 25 mL of YPD broth in a rotary shaker at 150 rpm. The cells were counted using a hemocytometer and, to induce capsule formation, suspended at 10^5^ cells/mL in capsule-inducing minimal medium (3 µM thiamine, 15 mM glucose, 10 mM MgSO_4_, 29.4 mM KH_2_PO_4_ and 13 mM glycine) following incubation for 72 h at 37 °C with 5% CO_2_.

### Sample preparation for SEM image acquisition

Capsule-induced cells were collected by centrifugation at 3,000 g for 5 min, washed three times in PBS and fixed (2.5% glutaraldehyde type 1 in 0.1 M sodium cacodylate buffer) for 1 h. Then, cells were washed in post-fixative solution (0.1 M sodium cacodylate buffer, 0.2 M sucrose and 2 mM MgCl_2_), and adhered onto coverslips coated with 0.01% poly-L-lysine, for 30 min. The coverslips containing cryptococcal adhered cells were dehydrated in solutions of graded ethanol (30, 50 and 70%, for 5 min/step, then 95% and twice 100%, for 10 min/step). Samples were subjected to critical point drying (Critical Point Dryer CPD030 - Balzers), mounted on metallic stubs, coated with a 15–20 nm gold layer, and visualized in a scanning electron microscope (Zeiss Auriga), operating at 5–10 kV.

### Image pre-processing and object identification

Based on the EBImage R package, we defined an automated segmentation pipeline capable of properly separating actual cell-associated pixels from background noise^12^. For preprocessing, all 11 images were primarily set to grayscale mode and 1024 × 724 pixels. Adaptive thresholding was performed using linear filtering. The local calculated background was removed from the original images for the construction of binary masks: in which 1 represents an object-associated pixel (foreground), and 0 represents the background, for each position in the image matrices. However, as the objects in these binary images often presented holes and blurred separations, they were propagated towards more lenient, fully filled binary masks through Voronoi tessellation^32^. Segmented objects with less than 1000 pixels were considered as obvious artifacts and hence assigned to the background.

### Feature extraction

The identified objects proceeded to feature extraction, which included basic (*e*.*g*. intensity mean and standard deviation), shape (*e*.*g*. area, max radius, mean radius), and moment (*e*.*g*. eccentricity) variables. Texture was analyzed using gray-scale co-occurrence matrices as in EBImage’s implementation of original Haralick features^12,33^. Variable names were coded as follows: *x*.*s*.*y*.*z* in which *x* is a placeholder, *s* is the feature source (*e*.*g*. binary mask or original image), *y* is the type (*e*.*g*. basic, shape), and *z* is the actual feature name (*e*.*g*. mean, area, radius). The feature type is identified as “s”, “b”, “m”, and “h” for shape, basic, moment, and Haralick features, respectively. Feature source varied among “0”, “a”, and “Ba” for binary mask, original images, and top-hat transformed images, respectively. Note that only translation- and rotation-invariant features were kept for model construction to avoid spatial biases - *i*.*e*. cells being classified based on position-related characteristics. The final data set was comprised of 811 cells and 54 features.

The identified objects were manually labeled as regular (N = 285), bald (N = 129), or spiky (N = 261) morphotypes. Borderline cells detected by the pipeline were labeled as unidentified when manual labeling was hampered (N = 74). Also, as the high-throughput image processing yields artifacts, these were also labeled accordingly and considered for modeling (N = 62).

### Random forest classifier

The labeled objects were then used to train a random forest (RF) model for morphotype classification. The optimization process was performed using the interface available from the Caret R package^23^. The RF algorithm chosen was the implementation from the Rborist R package, which allows for parallelism and fast optimization (within Caret) of two of the most relevant hyperparameters: minimal node size and number of randomly selected predictors at each split^24^. The optimal values were 9 and 10, respectively. The original data set was split into training and testing sets in a way that original class distributions were retained - using the sample.split function from Catools R package. All modeling optimization was carried out using repeated 10- fold cross validation (10 times) performed entirely on the training data set. Random Forests were constructed with 2000 trees each. The phantom capsular morphotypes were left aside as their total count was considered too low for model fitting (less than 3% of total cells).

### Statistical analysis

Non-parametric comparisons were performed using Kruskal-Wallis and Wilcoxon tests. Robust regression was carried out using M estimator as implemented in the MASS R package^34^. Benjamini-Hochberg procedure was used for multiple comparisons correction of P-values. An alpha level of 0.05 was used as significance threshold in hypothesis testing.

## Supplementary information

Supplementary Information.

## Data Availability

All code and raw data are available at https://github.com/giulianonetto/crypto_classification. The pipeline is capable of (i) identifying potentially cell-associated objects in the SEM-derived images; (ii) computing object-level features; and (iii) classifying these objects into their corresponding classes.
