# Supplementary material for: Scanning electron microscopy and machine learning reveal heterogeneity in capsular morphotypes of the human pathogen Cryptococcus spp
Source: Sci Rep. 2020 Feb 11;10:2362. doi: 10.1038/s41598-020-59276-w (PMC7012869; doi:10.1038/s41598-020-59276-w)
Supplement: Supplementary file 1 — Supplementary Information. [file 41598_2020_59276_MOESM1_ESM.doc]

**Scanning electron microscopy and machine learning reveal heterogeneity in capsular morphotypes of the human pathogen *Cryptococcus* spp.**

**William Lopes^1^, ^+^, Giuliano N. F. Cruz^2, +^, Marcio L. Rodrigues^3,4^, Mendeli H. Vainstein^5^, Livia Kmetzsch^1^, Charley C. Staats^1^, Marilene H. Vainstein^1^, Augusto Schrank^1, *^**

^1^ Centro de Biotecnologia, Universidade Federal do Rio Grande do Sul, Porto Alegre, Rio Grande do Sul, Brazil;

^2^ BiomeHub, Florianópolis, Santa Catarina, Brazil;

^3^ Instituto Carlos Chagas, Fiocruz, Curitiba, Paraná, Brazil;

^4^ Instituto de Microbiologia Paulo de Góes (IMPG), Universidade Federal do Rio de Janeiro (UFRJ), Rio de Janeiro, Rio de Janeiro, Brazil;

^5^ Departamento de Física, Instituto de Física, Universidade Federal do Rio Grande do Sul, Porto Alegre, Rio Grande do Sul, Brazil.

^*^Corresponding author

E-mail: [argusto@gmail.com](mailto:argusto@gmail.com)

**^+^**These authors contributed equally to this work.

**Supplementary Information (SI)**

**
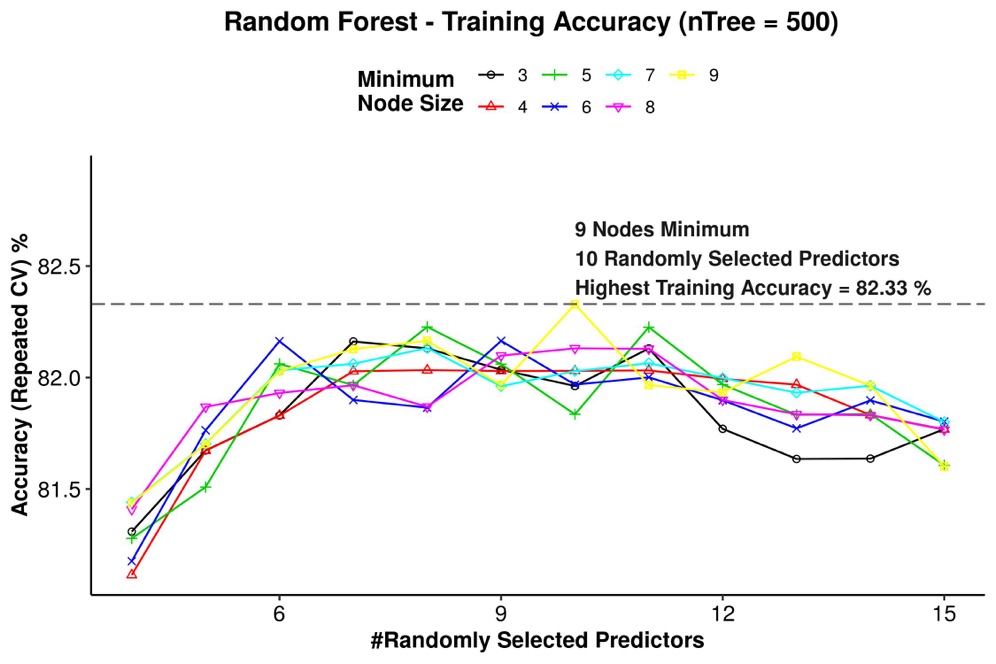
**

**Supplementary Fig. S1.** Tuning random forest hyperparameters. Accuracy estimations were generated from 10-fold cross-validation repeated 5 times. The training set comprised 75 % of all data. The best performance was achieved considering 11 randomly selected predictors at each split and having 9 as the minimum node size. Tuning procedure was performed using Rborist implementation from within the Caret modeling interface. The dashed line represents the highest accuracy point.

**
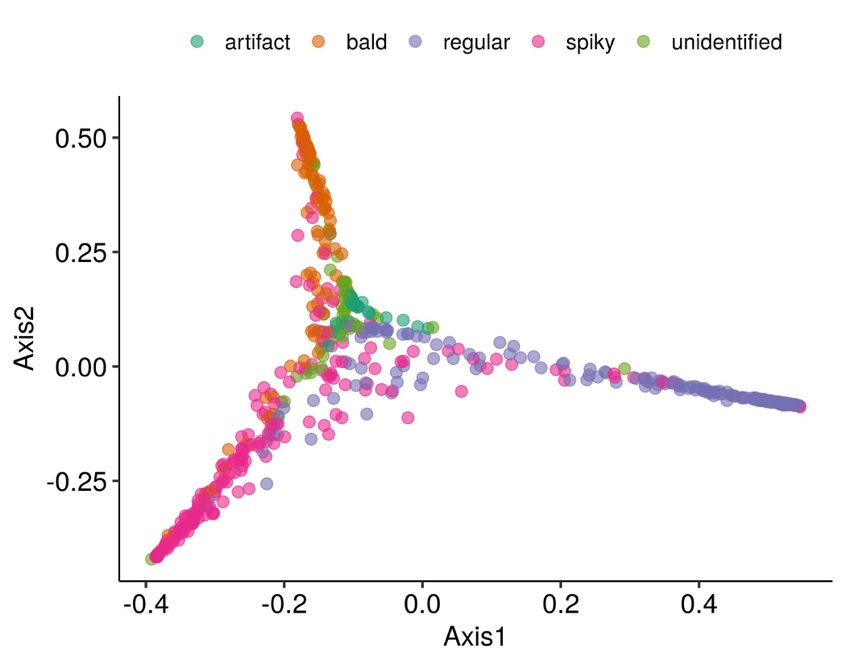
**

**Supplementary Fig. S2.** Principal Coordinate Analysis (PCoA) derived from sample proximities estimated by RF model. The random forest algorithm yields estimates of observations’ proximities. A distance matrix was constructed by subtracting these values from one, serving as input for PCoA (also known as Multidimensional Scaling). The star shape is typical of this analysis (proximity plot). While unidentified objects and artifacts seem gathered in the middle, cell classes tend to form groupings apart from each other. Here we only visualize two dimensions from estimated proximity values - while the model itself considers the actual feature space.

**
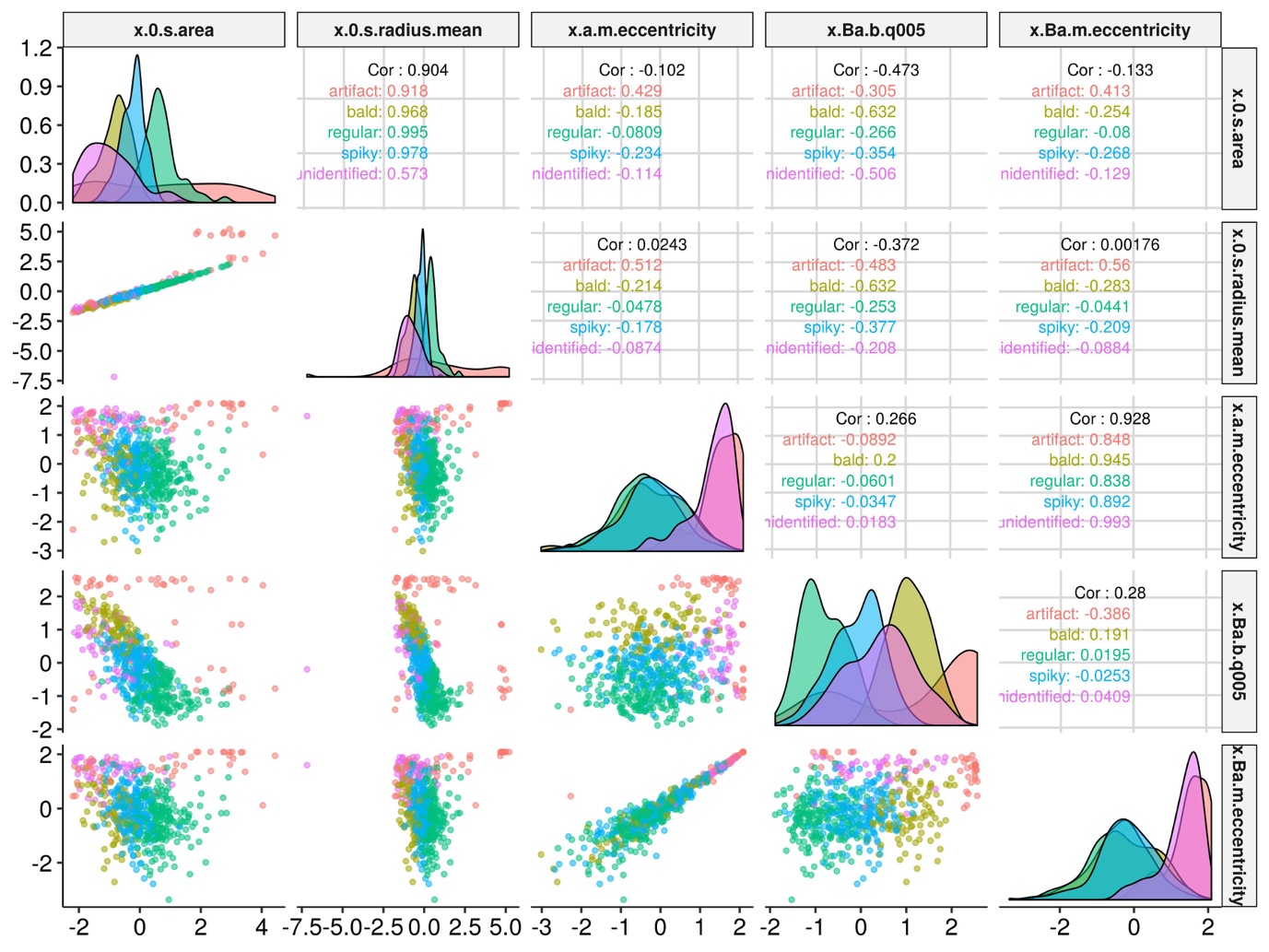
Supplementary Fig. S3.** Correlations among the five most relevant features and their distributions. As indicated by the mean decrease in the Gini index, the five most important features seem to include shape, moment, and basic characteristics. Note that x.Ba.b.q005, the 5-quantile of pixel intensities after top hat transformation, is the only basic feature among the top 10 variables - others include texture (Haralick) features. Expectedly, objects’ area and radius mean are highly correlated, which also happens between eccentricity calculated with reference images (x.a.m.eccentricity) and with the binary mask (x.0.m.eccentricity). However, the correlation values vary across classes. Additionally, notice that two-dimensional scatter plots fail to separate object classes completely - it is the features’ cumulative information that is used by the RF algorithm to make predictions.
